# Health Promotion for Childhood Obesity: An Approach Based on Self-Tracking of Data

**DOI:** 10.3390/s20133778

**Published:** 2020-07-06

**Authors:** Nazaret Gómez-del-Río, Carina S. González-González, Pedro A. Toledo-Delgado, Vanesa Muñoz-Cruz, Francisco García-Peñalvo

**Affiliations:** 1 Grupo GRIAL, Instituto Universitario de Ciencias de la Educación, Universidad de Salamanca; Paseo de Canalejas, 169, 37008 Salamanca, Spain; ngomrio@usal.es (N.G.-d.-R.); fgarcia@usal.es (F.G.-P.); 2 Grupo ITED, Computer Engineering and Systems Department, Universidad de La Laguna; Avda. Astrofísico Sánchez s/n, Physics and Mathematics Building, La Laguna, 38204 Tenerife, Spain; petode@ull.edu.es (P.A.T.-D.); vmunoz@ull.edu.es (V.M.-C.)

**Keywords:** child obesity, physical activity, user model, recommender system, UX, QS

## Abstract

At present, obesity and overweight are a global health epidemic. Traditional interventions for promoting healthy habits do not appear to be effective. However, emerging technological solutions based on wearables and mobile devices can be useful in promoting healthy habits. These applications generate a considerable amount of tracked activity data. Consequently, our approach is based on the quantified-self model for recommending healthy activities. Gamification can also be used as a mechanism to enhance personalization, increasing user motivation. This paper describes the quantified-self model and its data sources, the activity recommender system, and the PROVITAO App user experience model. Furthermore, it presents the results of a gamified program applied for three years in children with obesity and the process of evaluating the quantified-self model with experts. Positive outcomes were obtained in children’s medical parameters and health habits.

## 1. Introduction

Obesity is regarded one of the most problematic illnesses in developed countries [1]. Its consequences for life expectancy and quality of life are severe. Obesity is not merely an aesthetic problem, but represents a real social health problem, with significant short-, medium-, and long-term consequences. These impacts are not only physical, but also emotional and social, and directly affect the well-being of the population. Consequences occur at the individual, health, and economic levels. Thus, it has been postulated in a number of countries that obesity may represent an unsustainable burden on the health system. The problem of obesity is even more severe when it affects children.

Childhood obesity is the most prevalent chronic disease in developed and developing countries. The incidence of overweight and obesity among children has increased dramatically in recent decades. One of the most critical risks of childhood obesity is that being overweight in early childhood increases the risk of obesity later [2]. A rise in the likelihood of diseases [3] and obesity-related health risks has also been documented, including an increase in the incidence of type-2 diabetes among young people in recent years [4].

The problem of poor patient adherence can be a pervasive threat to health. The current approach to treating obesity often focuses primarily on weight loss. Self-motivation is essential for adhering to change; however, it can be challenging for individuals to adhere to a routine of exercise and physical activity despite knowledge that lifestyle change is the most effective method for long-term weight management. The use of wearable health monitors and predictive analytics techniques provides the basis for an excellent healthcare system [5].

In children, the reasons for not adhering to healthy routines involve problems related to adapting schedules, refusing to perform the treatment, and dissatisfaction with the results, among others [6]. Lack of adherence may lead to various problems related to the patient’s capacity to change their habits and to commit to the treatment. Furthermore, there is a gap in the literature regarding the effects of gamification in the long term [7]. As a result, we developed an educative gamified program, named PROVITAO, to promote healthy habits using motor play and exergames [8]. This gamified educational program enhances patient learning in a fun way using gamification techniques and aids in weight loss maintenance using games. Consistent with the available bibliography, observations of the program, in which children met with their peers and professionals, showed a strong attachment was made to the proposed activities during the programmed interventions, followed by a quick decay in interest to continue the activities on their own during the months following the program sessions.

A solution to the problem of adherence may be provided by technology. In recent years, the number of mobile apps available to facilitate therapeutic adherence has increased. Park et al. [9] carried out a study of the efficacy of mobile phone interventions and demonstrated that personalized feedback has positive effects on medication adherence. Other studies have also been carried out to evaluate the effectiveness of technological means to increase adherence to treatment [10,11,12], although it should be noted that some applications only remind the patient to take medication. In contrast, other applications have more content, such as educational interventions or rehabilitation exercises [13,14].

The popularization of devices that make it possible to continually measure different variables related to a person’s activities has led to the appearance of what has been termed the Quantified Self (QS) [15].

According to the Quantified Self Institute, the term “quantified self” embodies self-knowledge through self-tracking. Some common points of QS are data collection, the display of these data, and the cross-referencing of these data to discover possible correlations [16]. Measures about the self can be organic, bodily, behavioral, or environmental [17]. These data can be recorded daily (heart rate, respiration, hours slept) or even more frequently (blood pressure or weight, for example). Other data that can enrich the QS user model can be related to short-term and long-term information, such as the attitudes, behaviors, effects, and cognitive functions of users [18,19]. QS can involve the graphical presentation of the information and a response loop of reflection and self-analysis [17]. Recent studies have shown that behavior and physical exercises with activity supervision raise activity to reduce overweight or obesity [20]. However, high attrition rates after a number of months have also been reported [21,22]. Moreover, the implementation of self-tracking for health has also received criticism [23].

PROVITAO is a project, a technology ecosystem, and an intervention program for the promotion of healthy habits in children with obesity. Its scope is broader than the contribution made to this paper [7]. The PROVITAO App is one of the technologies of the mentioned ecosystem. The app is a closely related implementation of what is proposed in this paper. Although discussion of the development of the app is not an objective of this paper, herein the user experience model developed for the app is analyzed.

We propose a QS multidimensional model for children aged 6 to 12 years old to promote healthy habits by self-tracking of their daily activities. Several research problems emerge from this issue, namely:

- RQ1. What is an appropriate multidimensional QS model for promoting healthy habits in children?
- RQ2. How might a recommender system complement the QS model to help the user select the appropriate actions and the time to do them?
- RQ3. How can the User eXperience (UX) of using a QS approach for children be improved?
- RQ4. How does a gamified technology-based intervention impact the health of children?

Therefore, the main contributions of this paper are five-fold.

First, quantified-self practices in collecting and exploring personal data about their life activities are analyzed [24].

Second, we propose and describe a QS model of children with obesity. The purpose is to explore the potential change of behavior in children with better and healthier habits. Experts have previously validated the model in the context of the PROVITAO Project.

Third, the primary data sources for the QS model are also described. These sources include wearables, mobile phones, apps, activity logs, and sentiment analysis.

Additionally, a UX model of the PROVITAO App is included. This aspect is critical for our purpose to improve adherence to the intervention program. The model considers seven components: objective and subjective system aspects, user experience, interaction, user profile, contextual characteristics, and gamification.

Finally, an activity recommender system (ARS) is central to the use of the information in the models, and is activated during the interaction of the user with the system. It also includes the modules of action filtering, recommendation trigger, and action chooser.

Moreover, this paper also includes validation of the proposed models, and validations of the QS model and the gamified interventions are described.

The paper is organized as follows. In Section 2, we analyze the state of the art and related research on QS and its health applications. Then, in Section 3, we describe the technological solution we designed for this project, focusing on the QS model and the activity recommender system to promote healthy habits in obese children. Finally, we present the validation results in Section 4 and the conclusions in Section 5.

## 2. State of the Art

Wolf and Kelly defined the term quantified-self in 2007 which, according to Swan [25], can be defined as “any individual engaged in the self-tracking of any kind of biological, physical, behavioral, or environmental information, as a proactive stance toward obtaining information and acting on it.” This author also provided several examples of different quantified self-data, such as:

- Physical activities (distance, steps, calories, repetitions, etc.)
- Diet (calories consumed, satiety, fat, etc.)
- Psychological states (happiness, anxiety, depression, etc.)
- Mental and cognitive states (patience, creativity, reaction, memory, etc.)
- Environmental variables (location, noise, weather, etc.)
- Situational variables (context, time, date, etc.)
- Social variables (influence, charisma, status, etc.).

Swan (10) defined quantified self-tracking as the collection of information (physical, biological, environmental, or behavioral) that can be measured by an individual. The technologies should make it easy to use and access this information, and the data should be accurate and suitable for analysis. In addition, machine learning techniques should be employed to extract useful information [26].

There are several varieties of QS projects. In the sections that follow, we focus on those related to health.

### 2.1. Quantified Self for Health

QS is increasingly being used for healthcare with new methodologies and biometric data analysis [27]. In this environment, QS application has many advantages: self-healing, self-discipline, self-improvement, self-knowledge, etc. The main advantages are the positive effect of preventive medicine and enabling the patient and doctor to see updated information in real-time [28,29]. QS has been applied to health in several practices, including personal informatics. There are several health tracking devices, including accelerometers, pedometers, smartwatches, wrist-worn devices, wearables biosensors, clothing and wearable textiles, and smartphone applications [30,31,32,33,34].

Shin and Biocca proposed a study of the design and evaluation of technologies and QS applications to identify different means to encourage healthy user behavior [35]. Their results showed that confirmation and gratification (for example, satisfaction with technology use and enjoyment) and confirmed the importance of usability in the promotion of healthy user behavior. In their research, they observed that health feedback was more efficient when shown in text and comparative form, and that comparative feedback motivated users more than non-comparative feedback. Shin previously presented a study of a quantified-self user experience that employed wearable devices for health monitoring, which highlighted the importance of comparative feedback and of taking the characteristics and differences of users into account when designing strategies for improving healthcare by using self-monitoring or self-tracking devices [36].

Régnier and Chauvel conducted an interview study of the influences of various factors (social, cultural, and economic) on the use of self-tracking and fitness applications [37]. Their results demonstrated that users from lower milieus used fewer digital devices and participated less in social media (due to the cost of the devices, etc.), and therefore benefited less from a healthy lifestyle. However, the study results showed that, in terms of healthy behaviors, digital self-quantification devices are less important than their use.

Nevertheless, not all health tracking information involves automatic sensing derived from devices or biosensors. Sometimes other user information is needed, such as food consumption or physical activities undertaken, moods, and emotions. For this, apps and web platforms exist to support the collection of health data from users and provide insights via visualization [38]. Quantified-self measurements can be observational [39] or experimental [40]. In either case, it is necessary to identify specific user profiles and objectives of the application to design customized systems that adapt to and motivate the user depending on their goal [41].

Williamson [38] analyzed the digitized future of physical education, in particular, “how health tracking technologies promote new ‘bio-pedagogies’ of organic optimization based on data-led and algorithmically-mediated understandings of the body.” The author suggested the need for a greater focus on “how algorithmic systems are becoming embedded in emerging physical education technologies and pedagogical practices.”

Didžiokaitė conducted a study of the use of the MyFitnessPal app for dieting to improve fitness or sports performance, in which participants primarily used the technology with limited goals and little discipline because the objective of the majority of the users was to lower calorie intake immediately [42].

### 2.2. Wearables for Childen and Digital Biopedagogical Platforms

Various wearable trackers for children exist, providing a means for parents to track their child’s activity. These include AngelSense, Trax Family, Hereo, My buddy Tag, The Gator, Weenect, Ambertalert, Flashme Sidney; in addition, Omate x Nanoblock, Lineable Junior, and Kiddo are under development. The following are designed especially for fitness purposes: Garmin Vivofit Jr. 2, Fitbit Zip, Unicef Kid Power Band, Nabi Compete, X-Doria Kidfit, and Fitbit Alta HR.

In terms of health, in the educational field, the emerging area of portable physical activity monitors that combine tracking and biosensing capabilities with built-in algorithms to calculate and estimate health and fitness levels is being promoted to schools [43]. Many health monitoring applications for children are designed to encourage healthy lifestyles, diet planning, and physical activity. The popular features of applications related to children’s health include the concept of caring for virtual creatures and satisfying their dietary and physical exercise needs, often combined with various gaming and competition elements and online social networking platforms [44]. In the future, physical education pedagogies may appear in schools with the use of health monitoring technologies; for example, digital technologies, pedometers, and aptitude tests are already used in tests to monitor and control physical education [45].

From the perspective of global citizenship and responsibility, the Quantified Self Institute manages a vital research project, “Wearable Technologies for Active Living,” to develop activity monitors for children and a data platform to analyze and present the results. Its portable sensor device “makes children and parents aware of their physical activity.” In addition, the analysis of data obtained by users from the platform that accompanies the activity monitors is intended to be used in scientific research in which obesity awareness, behavior, and prevention play an essential role. As an academic research program, the Quantified Self Institute provides evidence of how children’s activity and physical fitness have become significant scientific enterprises.

Regarding gamified fitness, health monitoring is not only focused on metrics; it can also be fun and entertaining. Competitive and avatar-based health devices, applications, and platforms, such as Sqord represent the gamification of digital health among children. Gamification is the result of device and platform designers who persuade and guide their users towards correct behaviors using the psychology of game design. Designers have thus become medical influencers by using persuasive computer techniques [46,47]. The gamification of self-tracking can also allow for better surveillance [48].

### 2.3. Quantified Self and User Modeling

QS tools and devices for tracking and collecting data of healthy people open new opportunities for modeling users. The variety of data that can be collected automatically is wide: physiological states (for example, blood pressure), cognitive states (for example, stress), behaviors (for example, movements or an average number of hours of sleep), spatial context (for example, places visited), and social context (for example, people met or interacted with) [49].

This large amount of data collected from users’ daily actions and behaviors is not limited to the user’s web activity, as is typically the case in traditional user models [49].

The data obtained on user behavior can be used to create user models in the short, medium, and long term (18). For instance, Sarzotti et al. [49] proposed an Enhanced User Model (EUM) with short- and long-term data on four types of information:

- Attitudes: feelings, states of mind, and desires for a particular item, event, or tendency;
- Behaviors: activities that happen at a precise moment in time (like tasks or actions), or habits (series of repetitive acts);
- Emotions: moods (emotive trends over a prolonged period); emotions (over a short interval);
- Mental states: cognitive functions (for instance, memory, attention) regarding an instant and cognitive skills associated with their performance over time, such as an increase or decrease of memory health or spatial orientation.

Therefore, in this paper, we describe a QS proposal for creating a short-, medium-, and long-term user model that focuses not only on cognitive functions, attitudes, behaviors, and emotions, but also on physical and healthy performance activities.

## 3. Study on Enhancing Healthy Habits in Children

### The PROVITAO Project

The recently developed PROVITAO project is a crucial precedent for the QS proposal described in this paper. Relevant details of the project are described here before the QS model is then detailed. The PROVITAO project aims to assist in the treatment of obesity in children, improve health, and prevent disorders in adulthood, as described in [7].

The methodology applied in the project was a quasi-experimental, longitudinal, and prospective three-year study. It consisted of two phases, with each phase having a control group (children with obesity who do not participate in the intervention program) and an experimental group (children with obesity participating in the intervention program). This program was carried out in its first year at the University Hospital of the Canary Islands (HUC), located in the province of Santa Cruz de Tenerife, Autonomous Community of the Canary Islands, Spain. The target population was children diagnosed with obesity/diabetes type II seen in the pediatric outpatient clinics of the HUC. In the second year, the study was carried out in the public schools of the District of La Laguna, Tenerife, Spain. The target population was the students enrolled in these participating centers who suffered from obesity.

The sample comprised 45 children aged between 6 and 12 years (25 girls and 20 boys). This sample was selected using an inclusion criterion of a diagnosis of childhood obesity (Body Mass Index (BMI) > Percentile (PC)95; unit of measurement kg/m^2^), and, divided into experimental and control groups. The inclusion and exclusion criteria for the sample selection were as follows:

- Inclusion criteria:
  * Children aged between 6 and 12 years with obesity, using as the criterion a BMI higher than PC95.
  * Children attending HUC’s pediatric outpatient clinics (Phase 1).
  * Students enrolled in the public school participating in this project (Phase 2)
- Exclusion criteria:
  * Children who did not have the necessary networking technologies at home (computer and Internet) and television (experimental group only). The project provided the remainder of the technological tools required for the intervention at home (Kinect sensor, Wii console, and Wii balance board) and in the group sessions.
  * Children whose parents did not wish to participate in the project.
  * Children with cognitive impairment preventing them from participating in the project.
  * Participation during the preceding 12 months in a clinical trial.

The sample selection method was as follows. In Phase 1, children who attended the hospital’s pediatric consultation for the first time and met the inclusion and exclusion criteria were chosen. They were invited to participate at the consultation, and the objective of the study was briefly explained to them. If they agreed to participate, the doctor provided the research team with the first data collected at the medical consultation, namely, confirmation of an obesity diagnosis in the child and contact details. In addition, parents were given an information document about the project and a small questionnaire used to identify the technology networks used in the home. Later, in an information meeting with the research team, informed consent was given to participate in the control or experimental group, and any doubts about the research were clarified. In Phase 2, the physical education teachers of each school were asked to record the weight, height, age, and sex of the students in the 3rd, 4th, 5th, and 6th grades of primary education, providing the research team with the anonymized data (the school assigned each child a number and only the school had the correspondence between the number and the identification of the child). Those children enrolled in that school, who met the criterion of Body Mass Index (BMI) higher than PC95, and whose ages were between 6 and 12 years, were chosen.

Subsequently, the school was informed that the children had been selected based on their BMI, and invited to a meeting where the objective of the study was briefly explained, and they were given an information document and a document on which to indicate informed consent.

The conduct of this work followed the ethical guidelines and principles for medical research involving human subjects, as set out in the Declaration of Helsinki adopted at the World Medical Association (WMA) Assembly in 1964, and in the latest update in 2004. Therefore, the knowledge and approval of the students’ parents or guardians were ensured. In addition, the research was approved by the ethical committee of the Hospital Universitario de Canarias (approval code: CEIBA2020-0410).

**Figure 1:**
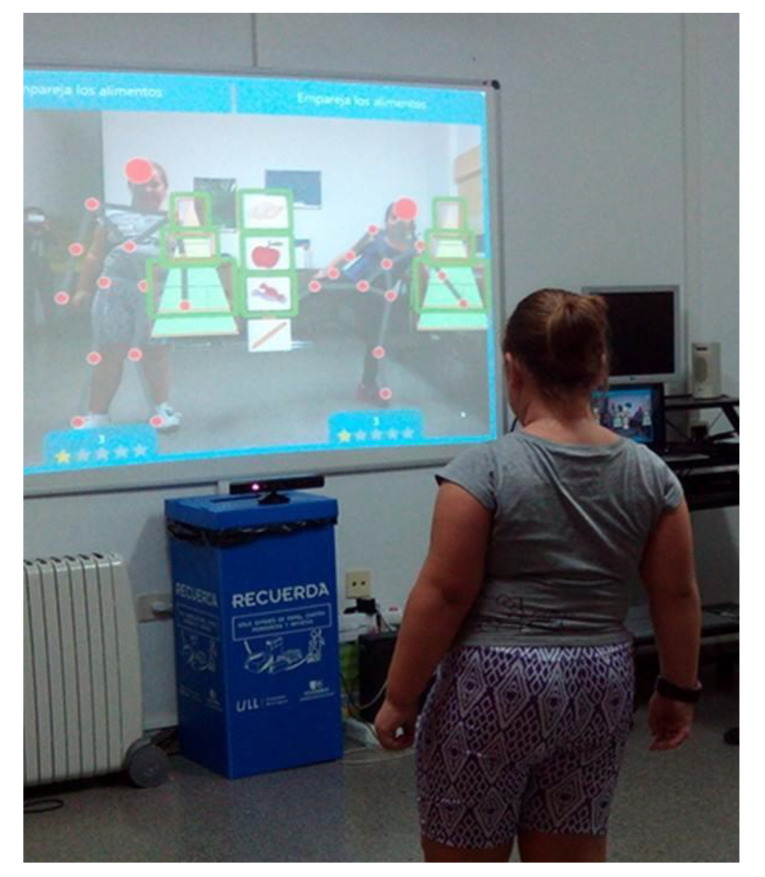
A patient of phase 1 of PROVITAO playing with TANGO: H and using the Geonaute Onmiles 600.

**Figure 2:**
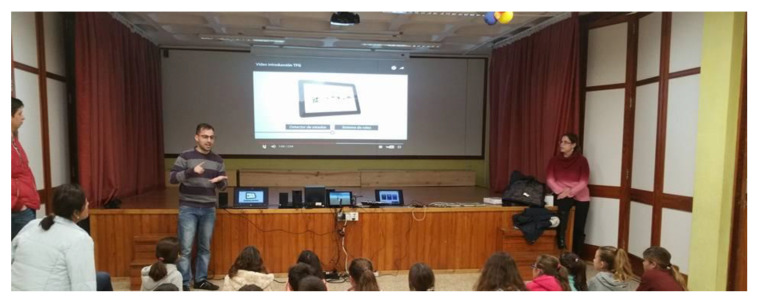
Children in phase 2 of PROVITAO using the provided applications.

We developed different serious games and apps for educational intervention to promote healthy living (some of the created resources are available at the website of the project [8]). One of these educational gamified tools and games was TANGO: H, using Kinect, which allowed the creation of different types of physical and cognitive exercises (43). Low-cost sensors (heart rate monitor and accelerometer) were used. In particular, we opted for a pulse and accelerometer wearable type wristwatch and belt from the brand Decathlon (Geonaute Onmiles 600). This type of commercial sensor provides its own analysis software. To study the potential of commercial sensors controlled by the provided software, a sensor integration library was designed and implemented to create interfaces and biometric signal records, allowing access to the data from commercial sensors. Finally, we created the PROVITAO App for smartphones and tablets, and a portal to gamify the activities performed by the children, with weekly missions that earned points, prizes, etc. The intervention was carried out in several phases (See Table 1, Figure 1 and Figure 2).

We defined a QS user model and other models to interact with the user due to the large and varied data sources used in this project (biometrics, anthropometric, interaction logs, tests, medical reports, etc.). Thus, in the following section, we describe the proposed models and data sources developed for the PROVITAO project.

## 4. Multidimensional QS Model for Promoting Healthy Habits

The main objectives of this paper are to describe a QS model, its data sources, a new architecture for an activity recommender system, and a UX module, all intended to enhance the adherence of children with obesity to a healthy lifestyle after an intervention stage. To accomplish our goals, the architecture includes the following components:

- Data sources (DS): We propose gathering data from multiple sources of information, including wearable sensors, mobile sensors, applications, digital activity records, and sentiment analysis applied to written communications.
- QS user model (QSUM): We define a QS user model that is continuously updated using the information mentioned above. The QS user model includes variables grouped into several aspects, such as attitudes, behaviors, emotions, and cognitive functions.
- Activity recommender system (ARS): Given the QS user model and the contextual information gathered about the user, a recommendation can be selected at any time from a predefined set of actions. To build the recommendation system, advanced data mining and machine learning techniques are applied [50]. The actions include engaging in a particular sport, participating in an active game, learning and reinforcing knowledge about healthy habits, and motivation enhancement tasks.
- UX Module (UXM): We propose a design for motivation enhancement tasks, although this design is itself an open research problem. It has been shown that adults who have access to data collected during their physical exercise sessions improve their performance and adherence. Consequently, we propose a means to present progress data to children to maximize their response. Due to the large and varied sources of data used in this project (biometric and anthropometric data, interaction logs, tests, medical reports, etc.), we define a QS user model and other models to interact with the user.

### 4.1. Data Sources

**Figure 3:**
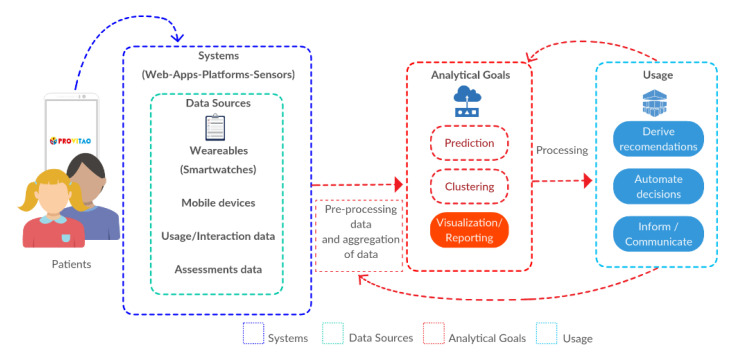
Main elements for making QS PROVITAO data relevant.

Data relevant to this study can be provided by multiple sources, including wearables, mobile phones, apps, activity logs, and sentiment analysis. Figure 3 shows the different data sources and systems, and the means of pre-processing and processing the data to make it relevant to the user.

PROVITAO manages diverse data from different aspects related to a patient with obesity. Table 2 shows the data grouped into different categories, such as player profile, emotions, behaviors, UX, social, diet, medical reports, anthropometric data, healthy lifestyle habits, and other relevant data.

### 4.2. QS User Model (QSUM)

**Figure 4:**
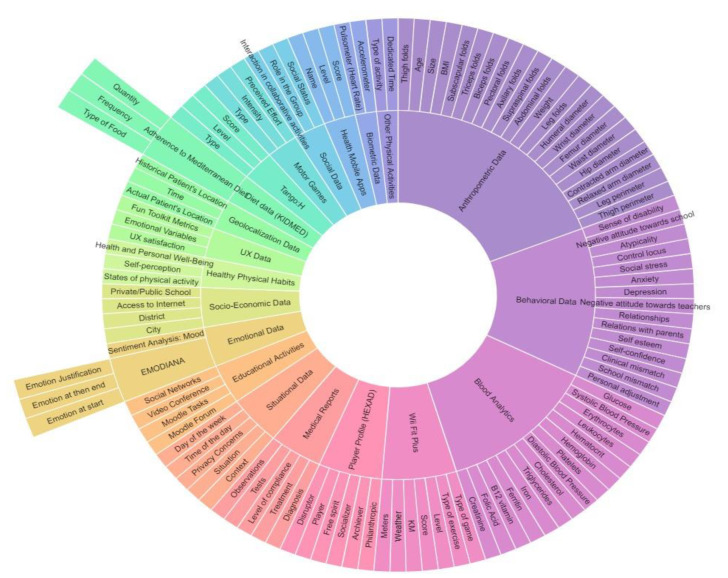
Data and aspects considered in the QS PROVITAO user model.

Our goal is to build a user model based on QS to be applied to obese children and to explore the potential for changing behavior toward healthier habits in the medium and long term. We also aim to enhance the QS through gamification. This user model contains information on attitudes, behaviors, emotions, learning, and health, and is supplemented with data collected from activities carried out in the real world using biometric devices, and data related to the user experience and gamification (Figure 4).

Thus, Figure 4 presents the different instruments, types of measurements, and data that we considered for the QS User Model.

Data regarding users came from medical reports (diagnosis, treatment, level of compliance, tests, and observations). These data were measured periodically.

Other types of measurement were provided by blood tests (systolic blood pressure, diastolic blood pressure, erythrocytes, leukocytes, hematocrit, hemoglobin, platelets, glucose, cholesterol, triglycerides, iron, ferritin, B12 vitamin, folic acid, creatinine, etc.). These measurements were taken at different moments of the intervention (pre-/mid-/post-).

Regarding anthropometric data, we measured age, weight, height, BMI, subscapular folds, triceps folds, biceps folds, inner folds, axillary folds, supraspinal folds, abdominal folds, thigh folds, leg folds, humeral diameter, wrist diameter, femur diameter, waist diameter, hip diameter, contracted arm diameter, relaxed arm diameter, leg perimeter, and thigh perimeter. These data were also taken at different stages of the intervention (pre-/mid-/post-).

We also collected biometric data from sensors, specifically from pulsometers (heart rate) and accelerometers (steps, speed, distance, pace) during session interventions.

In addition, we also considered the geolocalization data of the patient’s physical location, including time points and an individual patient’s location history.

Emotional data is another critical dimension of our QS User model. We collected these data per session (at entrance and exit) through the instrument EMODIANA [51].

Regarding the behavioral data, Behavior Assessment System for Children (BASC) was selected [52], which allows collection of different physiological measures, such as negative attitude toward school, negative attitude toward teachers, atypicality, control locus, social stress, anxiety, depression, sense of disability, relationships, relationship with parents, self-esteem, self-confidence, clinical mismatch, school mismatch, and personal adjustment. In addition, we included measurements relating to diet using the KIDMED questionnaire [53]. KIDMED provides data relating to adherence to the Mediterranean diet, namely, types of foods, frequency, and quantity. Moreover, data on healthy physical habits were considered in our model, such as states of physical activity, self-perception of motor competence, the usefulness of physical activity or sports, and data about health and personal well-being.

These data from BASC, KIDMED, and healthy physical habits were collected at different moments of the intervention (pre-/mid-/post-).

During face-to-face group sessions, we gathered data regarding the social dimension of gamification, such as the level of interaction in collaborative activities/games, the group’s role, and social status. User experience data were also considered, such as UX satisfaction (i.e., included in TANGO: H) and the emotional variables using the Fun Toolkit Metrics.

Before the intervention phase of the project, we determined the characteristics of the study participants using the HEXAD instrument. Thus, we classified our users into different players (i.e., philanthropic, achiever, socializer, free spirit, or disruptor). Other data collected at this phase were related to socio-demographic information, environmental data (family and school), and personal characteristics (age, sex, educational level, attitudes, and motivation).

During the intervention, information related to the situational data and user preferences, and to different activities carried out in the project, was gathered. These included active videogames (TANGO: H); exercises with the WII Fit Plus (i.e., aerobics, balance, toning, yoga, exercise plus); type of exercise (i.e., hula hoop, bike ride, land on white, tightrope, warrior, tree, Zazen, etc.), level, and score; motor games (the type of motor games, intensity, perceived effort); mobile health apps (i.e., the name of the game/app, level reached, score); and other educational activities (with time spent) in PROVITAO platforms (i.e., Moodle, videoconferencing and social networks, and other physical activities developed by children, such as type sports, dancing, running, and walking).

### 4.3. A Recommendation System to Complement the QS Model: The Activity Recommender System (ARS)

**Figure 5:**
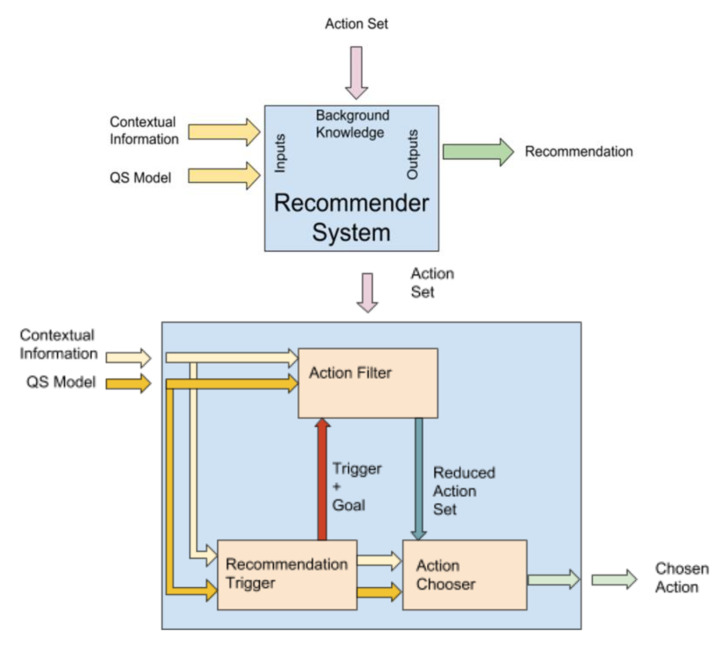
General model and sub-models of the recommendation system.

As described earlier, given the QS model and the contextual information, the recommendation system provides a recommendation from a predefined set of actions at any time. Formally, it can be defined as a purely abstract system with two inputs, one output, and background knowledge, as shown in Figure 5. However, the system itself might be complex because of the nature of the information involved. The recommendation system should be divided into modules that are able to work independently of one another.

The architecture of the recommendation system is based on the interaction of three different components: (a) the recommender trigger, (b) the action selector, and (c) the action filter (Figure 5).

#### 4.3.1. Recommendation Trigger

The recommendation system’s success depends on appropriately selecting a time at which recommendations are provided to the user. This module explicitly targets this issue.

An action can be triggered by various aspects:

Time and schedule: Depending on the time and the schedule of the users and actions, some actions are required to be scheduled. Reminder actions, weekly results summaries, scheduled physical activity sessions, etc., can be triggered within this aspect.
Location: When the user visits specific locations, an action can be triggered. For example, if the user is walking near a sports center that offers activities the user might be interested in, a reminder can trigger this location.
User model update: If the user’s QS model changes, an action can be triggered. Clear examples are the duration since the last physical activity session or time spent at a sedentary activity. When this time reaches a certain threshold, an activity recommendation is triggered.

The result of the recommendation trigger is not only the trigger event itself, but also the trigger goal. The trigger goal is a composition of the variables that influenced the trigger, and, should be the chosen action’s objective.

#### 4.3.2. Action Filter

The action recommendation will strongly depend on the user context. Not all actions may be appropriate because a natural set of constraints might exist beforehand. This context awareness approach to recommendation will ensure that only actions that fulfill some previously designed conditions are chosen. The approach to the action filter was designed based on an automated planning approach. Each action from the action set is linked to a set of preconditions and a set of goals. The set of preconditions is aligned with the aspects of the QS model and the contextual information. The QS model includes objective aspects, subjective aspects, and user profile features. Contextual information includes environmental, socio-economic, and situational data. The situational data includes not only the location of the user, but also timing and schedule information.

The preconditions are explicitly expressed as binary clauses built from the available variables. The set of goals for the action indicates the consequences the action might have on the user. Actions might seek to improve the physical training of the users, change their mood, improve self-awareness, reinforce some cognitive ability, etc. Only the actions that satisfy requirements, given the contextual information, QS model, and trigger goal, are sent to the action chooser module. Actions might be sent individually, or as an ordered group of actions, called a plan. A plan is designed as a concatenation of actions to be executed in a specified order. The selected order guarantees that when the action is executed, the preconditions have been fulfilled, given the consequences and the initial conditions of the previous action.

#### 4.3.3. Action Chooser

Once the recommendation is triggered, and the action set is filtered, a simplified version of the original recommendation system problem is addressed using the action chooser. Any of the filtered actions should be suitable for the user, and the time for the recommendation should also be appropriate. However, not every action is equally beneficial for users. Therefore, randomly choosing an action from the filtered action set is not an acceptable solution. The problem domain is significant, as is the number of dimensions, and therefore this does not seem appropriate for a content-driven approach to a recommendation. Even after the problem’s dimensions are reduced, the number of features to consider is sufficiently large to suffer from the curse of dimensionality, given the limited expected volume of the platform’s initial users. A traditional means of approaching such a recommendation is to build an expert system. The knowledge from professionals in the domain can be used to formulate rules to apply to the recommendation. One example of these systems is the fuzzy logic recommender, which encapsulates expert knowledge into a set of rules and a non-binary truth function. However, our proposed action chooser does not rely on some previously hardwired expert knowledge in the form of rules; on the contrary, a self-regulated learning algorithm is used instead. Based on a mix of content-based recommendations and collaborative filtering with learning, a reinforcement learning (RL) approach is used. Specifically, a simpler version of RL called the “Multi-Armed Bandit Problem” approach is implemented. The Multi-Armed Bandit Problem solution chooses the recommendation based on the expected output of that action in the current context. However, since, as previously stated, the dimension of the problem is large, it is assumed that the expected output for that specific context is merely a coarse approximation. More precise information about the expectation can only be retrieved after the experience of selecting different actions in the same context. The Multi-Armed Bandit Problem approach minimizes the number of incorrect recommendations that might be provided by the system before correcting its behavior. However, at the same time, a valuation of the results from the recommended action must be gathered from the user or the action to close the loop for the learning system.

#### 4.3.4. Recommendation Examples

Some examples of the types of recommendations in the form of actions, challenges, events, and reminders that the system considers are:

Healthy food restaurants and meals: At lunch or dinner time, the recommendation could be for a restaurant with a balanced menu based on your current location, or for a balanced meal at a specific restaurant.
Healthy locations: When the system detects that the user is enjoying free time, it recommends places to visit nearby that have health benefits for the user.
Physical activities: At the scheduled time for physical activity, a kind of activity is recommended, given the user preferences and the surrounding locations.

### 4.4. UX Model Using a QS Approach

The key to any recommendation system is the algorithm that makes recommendations based on the user’s data [54]. Thus, a large part of the research on recommendation systems has been devoted to creating and evaluating the best algorithms [55]. The better the algorithm, the better the recommendations it makes, and the better the UX will be in terms of the satisfaction and effectiveness of the system. Pu, Chen and Hu [56] evaluated the aspects related to recommendation systems from the user’s perspective and presented a model named ResQue (Recommender System’s Quality of User Experience) [57]. ResQue has four aspects: perceived system qualities, user beliefs as a result of perceived qualities, user subjective attitudes, and user behavioral intentions. However, other factors influence the user experience in recommender systems, such as situational and context factors, personal aspects, or privacy concerns. Knijnenburg et al. described a framework for UX in recommender systems, which they divided into objective and subjective aspects that influence the user experience [58].

**Figure 6:**
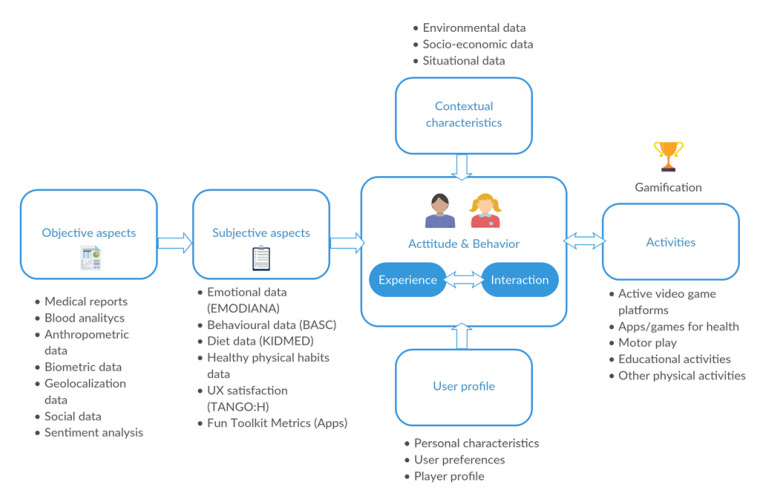
Components of our UX model for the PROVITAO recommendation system.

In the case of our UX, we considered for recommendation systems those aspects of QS that have not yet been thoroughly researched. Thus, we proposed a particular UX module that integrates the user experience point of view into the activity recommendation system. Adapting the model proposed by Knijnenburg et al. [59], our UX module considers six components: (a) the objective systems aspects (i.e., algorithms, biometrics, medical aspects, etc.); (b) the subjective system aspects (i.e., user perceptions of the objective aspects, subjective assessments of emotions, healthy habits, diet, etc.); (c) user experience (i.e., evaluation of the different parts (activities, systems, etc.) of the program); (d) interaction (i.e., interactions with different systems and platforms); (e) user profile (i.e., personal characteristics, player profile, preferences); and (f) contextual characteristics (i.e., environmental, socio-economic and situational data). In addition, we added a new module activity—gamification—to the model (Figure 6).

### 4.5. Gamified Technology-Based Intervention: The PROVITAO App Prototype

The above discussion presents the main contributions of this paper, which are related to the QS data set models. We now present a brief description of the PROVITAO App and its main functionalities. This web app was developed as a prototype to implement the different data models needed for the project, considering the varied data sources, instruments, and activities. This prototype was one of the tools developed as part of the PROVITAO project, however it was not used with the full intervention. Thus, the PROVITAO App was designed as a prototype of a progressive web app with a modular structure divided into the main roles or features to represent different patient therapy environments:

Clinical support. Allows therapists to design an appropriate protocol for each patient, including follow-up, the activities experienced by the patient, and the control of their effects.
Support at home. Allows communication between patient and therapist, enabling data of the process to be obtained and contributing to the child’s health education.
Mobile support. Facilitates the child’s access to advice and therapeutic instructions, and carries out the different weekly activities to achieve a series of rewards, making the treatment attractive.

**Figure 7:**
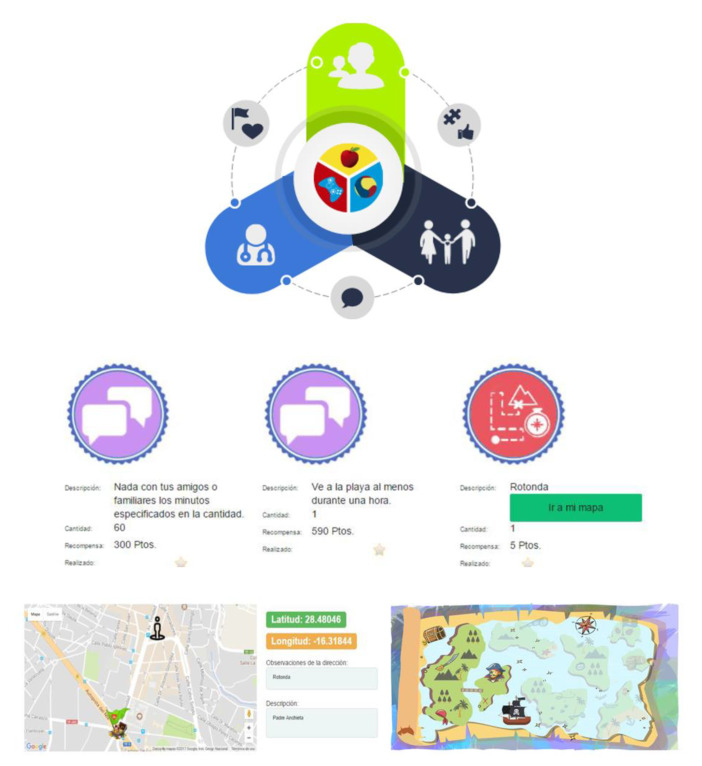
Roles in the PROVITAO App and examples of recommendations for three activities to undertake in a week, including description, time, and rewards, and the geolocalized activity “treasure map” with different islands to unlock.

This web app maintains communication between both parties, although each has specific functions (Figure 7). Thus, doctors can oversee monitoring of patients, advising families, and establishing weekly activities to encourage children to acquire healthy living habits. Children comply with these activities as part of their treatment. The activities provide children with points and rewards based on a pirate theme that ends with a final prize as they progress through a treasure map. With these small missions and games, children can remain active in the game, thus, not only complying with the treatment, but also implementing the knowledge they have acquired to date. Furthermore, the families’ work is highly decisive since the children are expected to imitate the people around them. Therefore, parents can also visualize their child’s evolution on a weekly basis, learning from doctors, nurses, and nutritionists, with the aim of integrating the child into the project quickly and comfortably.

In the next section, we present validations of the project. First, we present the validation of the QS model, followed by the usability of the prototype of the PROVITAO App, and, finally, the effectiveness of the gamified educational intervention in terms of learning and adherence to healthy habits.

## 5. Validation

In this section, we present the validations undertaken of this work. First, we describe the validation undertaken by the experts of the QS model; then, we present the validation of the user experience of the app; and, finally, we present the validation of the gamified technology-based intervention impact on the health of children.

### 5.1. Validation of the Multidimensional QS Model

The data sources of our QS model were validated by a convenience sample of 15 experts (eight female and seven male) from different areas of the PROVITAO project (medical, psychological, computer science, leisure-emotional, interactivity, health education) during iterative meetings. The experts were recruited based on their close relationship to the project and the relevance of their knowledge and area of expertise. These iterative meetings were held during the first six months of the project. The method used was a focus group, and a total of six sessions were conducted, organized into three phases (two meetings each phase, the first to propose and the second to refine).

The first phase’s goal was to decide which instruments to use with the target group (control group and/or experimental group or family). The second phase’s goal was to define the variables to be measured, their relationship to the research questions, and the systems and devices that could be used to collect data. The final phase in the definition and validation of the QS model involved organizing all of the data into different components, and validating them as inputs and outputs of the system. In the final phase, all of the experts shared their preliminary proposals to agree on the QS model’s final proposal.

The experts were grouped by areas of expertise to discuss and decide on the proposed QS model as follows (Table 3).

### 5.2. UX Validation

The user interface of the PROVITAO App was heuristically evaluated with a group of 10 experts (six men and four women) in the areas of computer science, health, and education, and from different countries, such as the USA, Ecuador, and Spain. The experts addressed a significant usability issue based on Nielsen’s established heuristics list (56). As a result, ten heuristics were evaluated.

Each team member independently performed each task on the website/app, documented usability problems and the heuristic infringements that took place, and rated each problem according to its severity. The usability problem severity rating was constructed as follows: 0—this is not a usability problem; 1—cosmetic problem; 2—minor usability problem; 3—major usability problem, requires fixing; and 4—usability catastrophe, must fix.

The chosen groups of usability findings were split into two parts: (a) negatives (Table 4) and (b) positives (Table 5). Five negatives and three positives are presented, associated with the specific issue and the heuristic(s) violated, and the severity of the issue is listed for each finding. Table 6 then shows the average user satisfaction for 82 heuristics evaluated by category.

### 5.3. Validation of the Gamified Educational Intervention

In this section, we describe a summary of the validation of the PROVITAO gamified program. The main goal was to validate the efficacy of a gamified educational program in minors with obesity. It was initially proposed that a criterion for inclusion of children in the second phase would be a type 2 diabetes diagnosis associated with obesity; however, it was challenging to find children admitted to hospital with both pathologies. This is because, since insulin resistance is detected, some medical treatments are actives; thus, the development of diabetes is stopped. Consequently, we studied the case of a child with obesity associated with metabolic syndrome and conducting a single case study [60].

We analyzed the results of questionnaires using SPSS 20.0 (Chicago, IL, USA). Then, we undertook a comparative analysis of the results obtained from the different data collection times (pre- vs. post-) and groups.

The responses of the children to the questions relating to health-related habits were analyzed and organized according to their self-perception of their motor competence, their opinion about the usefulness of physical activity or sport, their knowledge about healthy eating, and, finally, their perception of their health and well-being. Higher scores suggest healthier habits, better knowledge, and better satisfaction. The variables were distributed normally corresponding to the Kolmogorov–Smirnov test [61]. The similarity of the means in the baseline suggests that both groups showed analogous scores at the beginning of the study (see Table 7).

No statistically significant improvement between the control and experimental groups in physical activity behaviors was found; however, compared to the control group, the experimental group revealed significant progress related to knowledge about healthy eating over the long term. The interaction between the monitoring and group factor was not significant (F(2.50) = 2.582; *p* = 0.086; η2p = 0.094; *p* = 0.492), nor was there any significant group effect (F(1.25) = 0.503; *p* = 0.485; η2p = 0.020; *p* = 0.105). In contrast, there was a significant effect of the follow-up factor (F(2.50) = 28.647; *p* = 0.000; η2p = 0.532; *p* ≥ 0.999). After applying the Bonferroni adjustment to the pair analyses, it was observed that the scores over the long term were significantly different from those of the baseline (t(23) = 7.002; *p* ≤ 0.001; d = 1.2) and those of the immediate follow-up (t(23) = 6.234; *p* ≤ 0.001; d = 0.99). Examining the progression of the means considering both groups together, in both cases the greatest means in knowledge were found in the long term (Table 7).

In addition, no significant differences were found between the control and experimental groups or between the different collection times in the anthropometric measures (Table 8). As a reference value of childhood obesity, we used the standardized growth charts of the Faustino Orbegozo Foundation, which is recommended by the Spanish Association of Pediatrics [62]. These tables establish reference values as follows: for boys with an age of 8.5 years the median BMI is 17.69, the median height is 1.32 m, the median weight is 30.97 kg; for girls at the same age, the median BMI is 17.32, the median height is 1.30 m, and the median weight is 29.71 kg. In addition, the BMI value indicating obesity at the age of 8.5 is 23.5 for boys and 24.7 for girls; at the age of 9.5m, this value is 25.3 for boys and 25.6 for girls. Table 8 shows BMI values decreased after the intervention and were increased at follow-up, for both control and experimental groups. Hence, we cannot determine that the reduction was due to the intervention. However, in the single case analyzed, we found improvements in BMI, waist circumference, and body fat percentage [63]. Furthermore, we found significant differences between the control group and the experimental group in the BASC questionnaire (Behavior Assessment System for Children adapted from Reynolds and Kamphaus) [64]. For the analysis of the data, only those participants who completed the study and were old enough to provide a self-report were included. To compare the scores of the different participants, regardless of their age and the modality of the application questionnaire, we used only the indices of the self-report and standardized scores, that is, the average scores extracted from the application of the published scales, organized by age. According to the guidelines for the interpretation of the instrument, standardized scores greater than or equal to 60 (less than 70) in the difficulty scales, and less than or equal to 40 (greater than 30), in the adaptive scales are considered to be at risk; and scores with probable clinical significance are considered to be greater than or equal to 70 for the former and less than or equal to 30 for the latter.

For instance, in the experimental group, the feeling of responsibility and control over issues related to their lives increased. Nevertheless, negative sensations associated with social interactions decreased, as did the index of depression. Thus, we can observe the effectiveness of our intervention on healthy habits and in the promotion of the integral health of the children [65].

Concerning the game-based group intervention, the emotional study showed positive emotions related to the activities (participants, companions, games, learning). These results confirm the motivational significance of face-to-face meetings. The analysis of the emotions referred to by the participants at the entrance and exit of the face-to-face sessions provides an indicator of their motivation to take part in the intervention. However, we were particularly interested in the informative characteristic of the emotional impact of the intervention. Thus, to value the emotional effect of gamification, the game dynamics undertaken, and the training, we compared the emotions stated before the sessions with those stated afterwards. We examined all of the assessments taken throughout the interventions (12 sessions across the two phases). The emotions recorded with the EMODIANA tool were categorized into positive, negative, and neutral, and the mentioned aspects were reviewed using these categories. In all of the cases in which neutral emotions were reported at the beginning of the intervention, positive emotions were reported at the end. A share of 80% of the cases with negative emotions at the beginning were modified to positive emotions at the end; the remaining 20% finished with neutral emotions. A share of 4.3% of the cases in which positive emotions were reported at the beginning reported negative emotions at the conclusion, and 94.4% maintained positive emotions. Statistical analysis confirms that the differences in the distribution of the emotional categories are statistically significant both at the entrance (χ^2^ (2) = 195.571; *p* ≤ 0.001) and at the exit (χ^2^ (2) = 206.333; *p* ≤ 0.001). Each of the participants was asked to justify their reported emotion. These explanations were categorized by three judges following the criteria designed in the EMODIANA validation. The resulting categorization was submitted to concordance analysis for more than two judges using Fleiss’ Kappa. The first classification achieved a lower than desirable concordance, so some terms of the categories to be defined were clarified, and the independent categorization process was repeated for each judge. The resulting categorization was the one presented because it achieved a Fleiss’ Kappa index of k = 0.84; that is, an index value considered to be excellent (higher than 0.75) according to Fleiss (1981) [66], or very good (0.81 to 1.00) according to Altman (1991) [67].

Physical activity using motor play was designed to have moderate intensity (each session lasted 45 min), but children perceived effort to be easy or very easy (88.9%). However, the activity conducted was moderate or intense according to sensor data, which reached peaks close to 200 ppm (pulses per minute) in some cases. Therefore, although the activities demanded an average effort, for some children, the activities involved intense physical activity, which was expressed in their perceptions (8.2% reported some effort and 3.4% reported effort of higher intensity).

In the next section, we summarize the main findings, conclusions, and contributions of this paper.

## 6. Conclusions

This paper focuses on enhancing healthy habits in children using a QS model as the basis for an activity recommender system. To address the severe problem of child obesity outlined in this paper, an intervention program was designed including physical activities in groups, using active videogames, and reinforcing the practice of sports and knowledge of healthy nutritional habits, among other critical health-related activities.

On the basis of the intervention, and the design and development of the associated tools, a QS model and recommender systems were described. The following research questions were answered.

- *RQ1. What is an appropriate multidimensional QS model for promoting healthy habits in children?* Many different data sources can be integrated into a QS model in the context proposed. These information sources were identified and integrated into a single QS model, which was defined and validated by experts after an iterative process in which the experts were classified by areas of expertise.
- *RQ2. How might a recommender system complement the QS model to help the user select the appropriate actions and when to do them?* A recommender system for a sophisticated QS model cannot be designed as a single system. This paper proposes a recommender system supported by three different modules. One module selects when a recommendation should be made based on contextual and user information in the QS model. The second module filters recommendations to provide only appropriate actions for the user and the context, using an automated planning strategy. The third module selects the most appropriate action based on the action’s content, the user filtering information, and a learning strategy that minimizes the selection of mismatched actions. These three modules combine to answer, first, the question of what to recommend and, second, of when to recommend it, to thus help children improve their healthy habits.
- *RQ3. How can the UX of using a QS approach for children be improved?* The PROVITAO App is the framework in which our recommender system was embedded. Improving the UX of the system was considered to be crucial. Consequently, a UX model was explicitly proposed. The PROVITAO App was validated by experts who highlighted usability issues of the application, and evaluation of average satisfaction based on heuristic categories identified areas where the application UX could be improved.
- *RQ4. How does a gamified technology-based intervention impact the health of children?* We presented a summary of the validation of the gamified program using different instruments and dimensions. The PROVITAO project created a game-based educational program for healthy habits using active games developed by the research group, such as TANGO: H, and commercial video games (Wii Fit Plus and apps). The project was carried out with 45 children suffering from childhood obesity, plus their caregivers. Various technological artifacts (exergames, serious games, web apps, sensorial libraries, wearable devices, etc.) have been developed as part of this project. Results of the project have not shown a significant immediate or general improvement in measures such as participants’ body mass composition or percentage of body fat. However, there is evidence of a positive impact on the children, which might lead to a long-term positive effect. These changes include better knowledge about healthy eating, improved behavior and greater sense of responsibility, control over issues such as social stress and depression, a tendency to favor positive emotions after the intervention, and an improvement in the children’s free-time habits. Furthermore, in a single case study with obesity and type II pre-diabetes, there were observed improvements in measurements of BMI, waist circumference, and percentage of body fat, and these improvements were maintained over time. For future interventions, it is recommended that the positive effects of the mentioned measures are analyzed during the process, rather than only the ultimate measures and BMI.

Furthermore, from an economic perspective, the proposed intervention should be analyzed thoroughly before it is generalized for other programs, and the intervention’s economic analysis should be analyzed by estimating the program’s costs and revenues.

From the cost perspective, analysis of at least two types of expenditures—materials and infrastructure—and the necessary human resources should be conducted to determine affordability. The proposed program was designed to be replicated with a low cost. The space needed for the intervention is typically available at schools with a sufficient number of children. The materials required for the games and exercises are also inexpensive and usually available in schools’ sports facilities. The devices used by the children (wearables, sensors, videogames, etc.) were selected to be readily available technologies that are affordable for families. Although high precision sensors could marginally improve the recovered data, the information gathered using affordable and easily found home technology is sufficiently precise. From the perspective of human resources, PROVITAO program interventions have been supported by experts from a multidisciplinary research group: psychologists, nurses, medical doctors, physiotherapists, education and physical exercise experts, and computer science engineers. From this perspective, the cost of the sessions supported by these experts would make the intervention unaffordable in most cases.

Nevertheless, the number of professionals involved and their profiles were justified, not only to carry out the intervention, but also for validation from the perspectives of multiple disciplines. Once validated and appropriate infrastructure is permanently established for all sessions, the intervention is able to be carried out by two adults—one specialized in physically active games and the other with a technological profile. The sessions are programmed to take 4 h per week per group of 12 children. An extra hour per session is necessary for material preparation prior to the session and analysis afterwards. Therefore, 12 worked hours per intervention per week are required, which is considered reasonable. Pre-tests and post-tests require input from a nurse and a psychologist. Nevertheless, this support is only needed at specific moments of the intervention. It is expected that most children in this situation would already be under the supervision of medical doctors who could perform the timely actions required.

From the revenue perspective, the improvement in the quality of life of children with healthier habits is both evident and difficult to quantify. However, even from the public health perspective, the positive economic impact of the intervention is clear. Obesity and diabetes, like other chronic diseases, have an enormous impact on health systems, with patients requiring treatment for extended periods. Furthermore, future health complications can make suffers less productive, or even dependent. Decreased incomes, reduced productivity, and high costs of health interventions make it clear that, even without quantifying the reduction in the quality of life, early intervention in the lives of children is beneficial for society. Nevertheless, future work could show more precise results of the economic calculus expressed here.

The main contributions of this paper include the following:

- A technological proposal based on a quantified-self user model to treat and prevent obesity in children was presented. These components were described in this paper (i.e., DS, QSUM, ASR, and UXM).
- An extensive list of data sources and the main mechanisms for data processing are provided in the DS component, which primarily uses the data to recommend healthy activities. In addition, the categories of DS and relationships are described in the QSUM component.
- A new approach for an activity recommender system is presented with the following characteristics:
  - A modular design comprising a recommendation trigger, an action filter working as a content-based recommender, and an action chooser as a collaborative filtering recommender.
  - Actions and action plans are built using an automated planning approach.
  - Action recommendation mismatches are minimized using a reinforcement learning strategy.
  - A multidimensional, goal-driven recommendation trigger is used that relies on the user’s location, time, and schedule, as well as user model changes, as its primary considerations.
  - The UX model for recommender systems is extended from the one proposed by Knijnenburg (2012) to consider the QS particularities and gamification aspects.

Regarding the limitations of this proposal, its validation needs to be extended using a larger group of children, and evaluation of the recommender system’s efficacy and experience. However, a preliminary validation of the user interface of the PROVITAO App was presented in this paper, with an expert validation of the described QS model. The results indicate that the design, adapted language, and gamification are the strongest points of the proposal, while the main issues involve navigation controls, consistency and standards, profile personalization, error messages, and support. As a result, ongoing work on the system is aimed at solving these problems and improving technological solutions.

## Figures and Tables

**Table 1:** Description of the phases and goals of PROVITAO.

Phase	Goal
1: Pre-Intervention Phase	Permit application Fieldwork Preparation Cross-sectional simple sample study - Environment risk/protective factors - Unhealthy habits, level of knowledge - Obesity/overweight prevalence Target Population Definition data
2: Intervention	Intervention. 1st Quarter Gamified Process Group Sessions – Physical Activities + Tango: H Home Sessions – Wii Fit Plus (Session designs) – Mobile Apps Parent training activities Children Educational Program Intervention. 2nd Quarter Home Sessions – ICT Tools – Wii Fit Plus Vocational Project Contact with municipalities Orientation activities for parents and children Intervention. 3rd Quarter Execution of the vocational Project – ICT supported intervention – Videoconferencing – Tutored activities B-learning support and advice for parents and children
3: Post-Intervention Phase	Evaluate the influence of educational intervention. Technological tools effectiveness validation. Metric reevaluation after intervention phases.

**Table 2:** Type of measurements, data, and times when they are collected.

Type of Measurement	Data	When
Medical reports	Diagnosis Treatment Level of compliance Tests Observations	Periodical
Blood tests	Systolic blood pressure, Diastolic blood pressure, Erythrocytes Leukocytes, Hematocrit, Hemoglobin, Platelets, Glucose Cholesterol, Triglycerides, Iron, Ferritin, B12 vitamin, Folic acid, Creatinine, etc.	Pre-/Mid-/Post- Intervention
Anthropometric dataº	Age, weight, height, BMI, subscapular folds, triceps folds, biceps folds, pectoral folds, axillary folds, supraspinal folds, abdominal folds, thigh folds, leg folds, humeral diameter, wrist diameter, femur diameter, waist diameter, hip diameter, contracted arm diameter, relaxed arm diameter, leg perimeter, thigh perimeter	Pre-/Mid-/Post- Intervention
Biometric data from sensors	Pulsometer (Heart Rate) Accelerometer (Steps, Speed, Distance, Pace, etc.)	During session intervention/Diary
Geolocalization data	Patient’s physical location Timepoint Patient’s location history	Diary
Emotional data	Intervention (EMODIANA): -Input emotion (Positive, Negative, Neutral) -Exit emotion (Positive, Negative, Neutral) -Justification (Person, Structure, Context, Person-Structure, Person-Context, Structure-Context, Person-Structure-Context) Mood (sentiment analysis)	Per session (entrance and exit) Diary
Behavioral data (BASC)	Negative attitude toward school Negative attitude toward teachers Atypicality Control locus Social stress Anxiety Depression Sense of disability Relationships Relationship with parents Self-esteem Self-confidence Clinical mismatch School mismatch Personal adjustment	Pre-/Mid-/Post- Intervention
Diet data (KIDMED)	Adherence to Mediterranean Diet: Types of foods, Frequency, and Quantity	Pre-/Mid-/Post- Intervention
Data on healthy physical habits	States of physical activity Self-perception of motor competence and the usefulness of physical activity or sports Health and personal well-being	Pre-/Mid-/Post- Intervention
Social data	Level of interaction in collaborative activities/games Role in the group (gamification) Social status (gamification)	During session intervention
UX data	UX satisfaction (TANGO: H) Emotional variables Fun Toolkit Metrics (Apps)	During session intervention Home/Diary
Player Profile (HEXAD)	Philanthropic Achiever Socializer Free spirit Player Disruptor	Beginning of the intervention
Socio-economic data	City, District Internet access and connected devices School (Private–Public)	Beginning of the intervention
Situational data	Context Situation Privacy concerns Time of day Day of week	During intervention
Activities	- Active video game platforms: - *TANGO: H:* Type of Exercise, Level, Punctuation - *Wii Fit Plus:* Type of game (i.e., aerobics, balance, toning, yoga, exercise plus), Type of exercise (i.e., hula hoop, bike ride, land on white, tightrope, warrior, tree, Zazen, etc.), Level, Punctuation, KM, Weather, Meters - Motor games: Type of motor games, Level of intensity, Perceived effort - Health mobile apps/PROVITAO App: The name of the game, the level reached, Punctuation - Educational activities in PROVITAO Platforms: Moodle Forum, Moodle tasks, Video conference (group), Social networks - Other physical activities: Type of activity (Sports, Dancing, Running, Walking, Training, Playing, ...), time spent on it	Sessions designed for the group (weekly) Sessions designed for the home (weekly) Sessions designed for the group (weekly) Recommended activities to perform at home (weekly)
User Preferences	Favorite foods/drinks Favorite activities/games Favorite places	During intervention
Personal characteristics	Age Sex Educational level Attitudes and motivation	At the beginning of the intervention
Environmental data	Family environment School environment	At the beginning of the intervention

**Table 3:** Experts organized by area of expertise.

	Medicine (Biomedical, Physiotherapy, Pediatrics, Nutrition and Endocrinology)					Leisure-Emotion (Physical Education and Psychology)				Interactivity (Computer Science, Psychology)				Psychology and Education (Psychology, Health Education, Educational Technology)			
Expert	E1	E2	E3	E4	E5 *	E6	E7	E8	E9	E10	E11	E12	E13 *	E13 *	E5 *	E14	E15
Age	40	58	50	60	25	58	44	43	28	34	43	33	29	29	25	25	26
Sex	M	F	F	M	F	M	M	F	F	M	F	M	F	F	F	F	M
Title	ME	ME	ME	ME	NU	PE	PE	CS	PS	CS	CS	CS	PS	PS	NU	ED	CS

F: Female-M: Male; CS: Computer Science (PhD); PE: Physical Education (PhD); PS: Psychology (PhD); NU: Nursing (Master’s); ME: Medical Doctor (PhD), ED: Education (Master’s); (*) Experts in certain areas took part in more than one discussion group.

**Table 4:** Negatives and proposed solutions.

##	Problem	Heuristic (s)	Severity	Detail
1	Navigation	#1 #3	2	Users found difficulties in navigation and misunderstood the nav buttons. Lack of buttons to move between activities and to move back and forward.
2	Consistency and standards	#2	2	Some words or symbols can be confused. For example, the gamification “star” symbol does not appear in health professional profiles.
3	Personalization	#7 #3	1	Need more personalization features in user profiles.
4	Error messages	#5 #6	2	Signs usually accompany some error messages at the beginning that divert attention from the error message. The corresponding solution should accompany error messages.
5	Help	#10	4	Need to expand the help section and to write more instructions on some patient options. There should be a help section or, in its absence, instructions on each page, especially doctors.

**Table 5:** Positives.

##	Success	Heuristic (s)	Detail
1	Language	#4	Correct language adapted to children (in this case, patients).
2	Design	#8	Nice, adequate, and minimalist design. The information displayed correctly.
3	Gamification	#7	Good approach, especially the rewards map.

**Table 6:** Average user satisfaction for 82 evaluated heuristics, presented by category.

#	Category	Quantity Of Heuristics Evaluated	Average Satisfaction (Min:1–Max:5)
1	Visibility of system status	19	2.3
2	Match between system and the real world	8	2.3
3	User control and freedom	8	3
4	Consistency and standards	14	2.1
5	Error prevention	9	2.4
6	Recognition rather than recall	4	2.8
7	Flexibility and efficiency of use	11	1.9
8	Aesthetic and minimalist design	2	3.4
9	Help users recognize, diagnose, and recover from errors	3	1.9
10	Help and documentation	4	2.9

**Table 7:** Responses of children to the questions relating to health-related habits.

***Group and Statistical Means and (Standard Deviations) of the Difference in Mean Scores in the Children’s Baseline Questionnaires***						
	**Control** **N = 20**	**Experimental** **N = 25**	**F(1.43)**	**p**	**η2p**	**P**
Self-perception of motor skills and the usefulness of physical activity or sports	22.25 (3.40)	20.36 (5.60)	1.75	0.19	0.04	0.25
Feeding	27.00 (3.64)	26.40 (2.66)	0.41	0.52	0.01	0.10
Personal Health and Wellness	40.75 (5.73)	39.32 (7.03)	0.54	0.47	0.01	0.11
***Means and (Standard Deviations) of Questionnaire Rates Reported by the Two Groups of Children in the Follow-Up Assessments.***						
	**Short Term**		**Long Term**			
	**Control** **N = 16**	**Experimental** **N = 17**	**Control** **N = 16**		**Experimental** **N = 15**	
Self-perception of motor skills and the usefulness of physical activity or sports	22.69 (3.34)	20.59 (4.99)	20.81 (3.31)		19.80 (5.71)	
Feeding	27.00 (2.88)	28.71 (2.62)	29.56 (2.50)		30.87 (2.95)	
Personal Health and Wellness	40.25 (6.39)	40.18 (8.06)	40.63 (8.21)		38.80 (7.01)	

Note: After Bonferroni adjustment.

**Table 8:** Anthropometric measurements of control and experimental groups.

	***Control Group***											
	**Year 1**						**Year 2**					
	**Pre-Test**		**Post-Test ** **(Short Term)**		**Post-Test ** **(Long Term)**		**Pre-Test**		**Post-Test ** **(Short Term)**		**Post-Test ** **(Long Term)**	
	**Median**	**SD**	**Median**	**SD**	**Median**	**SD**	**Median**	**SD**	**Median**	**SD**	**Median**	**SD**
Age (years)	8.67	1.66	8.44	1.67	8.5	1.2	10.09	1.51	10.86	1.21	11.1	1.52
Weight (kg)	53	8.24	51.41	8.44	55	8.21	58.72	13.14	61.26	6.71	64.5	12.51
Height (m)	1.41	0.11	1.41	0.1	1.44	0.11	1.48	0.11	1.52	0.08	1.54	0.1
BMI (Kg/m^2^)	26.82	3.13	25.56	1.89	26.49	2.11	26.53	3	26.42	2.75	27.13	2.91
W/H Ratio	0.91	0.15	1.99	3.04	0.96	0.06	0.95	0.05	0.97	0.03	0.92	0.05
	***Experimental Group***											
	**Pre-Test**		**Post-Test ** **(Short Term)**		**Post-Test ** **(Long Term)**		**Pre-Test**		**Post-Test ** **(Short Term)**		**Post-Test ** **(Long Term)**	
	**Median**	**SD**	**Median**	**SD**	**Median**	**SD**	**Median**	**SD**	**Median**	**SD**	**Median**	**SD**
Age (years)	9.38	1.85	9.46	1.9	10.25	1.66	9.17	1.64	9	2.16	9.71	1.8
Weight (kg)	63.65	14.39	64.4	14.72	72.09	11.89	57.58	13.51	53.43	8.15	60.82	11.44
Height (m)	1.41	0.11	1.44	0.09	1.49	0.08	1.44	0.12	1.43	0.14	1.47	0.12
BMI (Kg/m^2^)	31.72	5.87	30.49	5.06	32.52	4.07	27.52	3.59	26.1	1.96	27.98	2.37
W/H Ratio	1	0.05	0.99	0.06	0.99	0.08	0.96	0.06	1	0.08	0.93	0.06

Note: Provisional results of the sample that passed the medical controls.
